# Safety and Performance of the Subcutaneous Implantable Cardioverter Defibrillator Detection Algorithm INSIGHT^TM^ in Pacemaker Patients

**DOI:** 10.3390/jcm13010129

**Published:** 2023-12-26

**Authors:** Kay F. Weipert, Srdjan Kostic, Timur Gökyildirim, Victoria Johnson, Ritvan Chasan, Christopher Gemein, Josef Rosenbauer, Damir Erkapic, Jörn Schmitt

**Affiliations:** 1 Department of Cardiology, Rhythmology and Angiology, Medizinische Klinik II, Diakonie Klinikum Jung Stilling, 57074 Siegen, Germany; rivtan.chasan@diakonie-sw.de (R.C.); josef.rosenbauer@diakonie-sw.de (J.R.); damir.erkapic@diakonie-sw.de (D.E.); 2 Department of Cardiology, Kantonsspital Aarau, 5001 Aarau, Switzerland; srdjan.kostic@ksa.ch; 3 Department of Cardiology, Lahn-Dill Kliniken, 35578 Wetzlar, Germany; 4 Department of Cardiology and Angiology, Medizinische Klinik I, Universitätsklinikum Gießen und Marburg, 35392 Giessen, Germany; 5 Department of Cardiology, Nephrology, Pneumology and Rhythmology, Klinikum Aschaffenburg-Alzenau, 63739 Aschaffenburg, Germany; 6 Department of Cardiology, Pneumology and Angiology, Medizinische Klinik II, Westpfalz-Klinikum, 67655 Kaiserslautern, Germany; joernschmitt@westpfalz-klinikum.de

**Keywords:** subcutaneous internal cardioverter-defibrillator, pacemaker, oversensing, detection algorithm, inappropriate therapy

## Abstract

Background: The use of the S-ICD is limited by its inability to provide backup pacing. Combined use of the S-ICD with a pacemaker may be a good choice in certain situations, yet current experience concerning the compatibility is limited. The goal of this study was to determine the safety and efficacy of the S-ICD in patients with a pacemaker. Methods: A total of 74 consecutive patients with a bipolar pacemaker were prospectively enrolled. First, surface rhythm strips were recorded in all possible pacemaker stimulation modes, to screen for T-wave oversensing (TWOS). Second, a S-ICD functional dummy was placed epicutaneously on the patient in the typical implant position. The same standardized pacing protocol was used as mentioned above, and every stimulation mode was recorded via S-ECG in all vectors. Results: In 16 patients (21.6%), programmed stimulation would have led to VT/VF detection. Triggered episodes were due to counting of the pacing spike(s), QRS complex, premature ventricular contractions, and/or additional TWOS. Three cases triggered in the bipolar stimulation mode. Oversensing was associated with lung emphysema and a reduced QRS amplitude in the S-ECG. Conclusion: The combination of an S-ICD and a pacemaker may lead to inadequate shock delivery due to oversensing, even under programmed bipolar stimulation. Oversensing cannot be sufficiently predicted by the screening tool in pacemaker patients. Testing with an epicutaneous S-ICD dummy in all vectors and stimulation settings is recommended in patients with pre-existing pacemakers.

## 1. Introduction

For decades, the implantable cardioverter defibrillator (ICD) has proven to be an effective and established therapy for the prevention of sudden cardiac death due to ventricular arrhythmias [1,2]. In contrast to the conventional transvenous ICD systems, the subcutaneous ICD (S-ICD; EMBLEM^TM^ Boston Scientific, Marlborough, MA, USA) and its lead are implanted entirely extrathoracically without direct cardiac contact. Its extrathoracic design is like that of the wearable cardioverter-defibrillator (WCD; LifeVest ^®^ Zoll, Pittsburgh, PA, USA), but the S-ICD is conceptualized to be a permanent solution for the patient. Unlike the transvenous systems, the S-ICD does not deliver anti-bradycardia or anti-tachycardia therapy. In exchange, it shows promising benefits with no risk of endocarditis and a presumed reduced risk for lead failure and fracture [3,4,5].

In patients with a complex anatomy who do not have the option of endovascular lead implantation, the S-ICD offers a potential alternative to epicardial lead implantation. As a consequence, the S-ICD is listed in the European Society of Cardiology guidelines as an alternative therapeutic option with a class IIa recommendation in patients with an ICD indication not requiring pacing for bradycardia, cardiac resynchronization therapy, or anti-tachycardia pacing, as well as a class IIb recommendation when venous access is difficult following the removal of a transvenous ICD due to infection or in young patients with a long-term need for ICD therapy [6]. In addition, the American Heart Association guidelines even refer to a class I recommendation for patients with a complex anatomy and venous access problems or for those at high risk of infections who need ICD therapy [7]. All cardiological societies base their recommendation primarily on two registries documenting safety and efficacy issues of the S-ICD [8,9].

In real life, there exists another patient cohort that, in our opinion, has not yet been thoroughly studied: patients who require pacing in the course of time after initial S-ICD implantation and patients who already have a permanent pacemaker prior to S-ICD implantation with difficult venous access. Both scenarios lead to a combination of two potentially life-saving devices that are not coordinated. Pacemakers can be either single- or dual-chamber devices and stimulation may be performed in unipolar or bipolar ways. Patients may be pacemaker-dependent or need only intermittent pacing intervention. However, we have learned that pacing spikes, particularly with unipolar pacing, can mislead the detection algorithm not only of implanted defibrillators and external defibrillators (AEDs) but also of the wearable defibrillator (WCD) [10,11]. Only small case series hint at the rather tangential role of device–device interactions between S-ICD and other transvenous intracardiac devices [12,13].

Misleading the detection algorithm can result in inadequate shocks or prevent life-saving defibrillation when ventricular tachyarrhythmic events are present. Thus far, there is one case report of inadequate shock delivery due to quadruple counting in a patient in whom a unipolar pacing spike misled the S-ICD detection algorithm (INSIGHT^TM^) [14]. Therefore, the aim of our prospective observational study was to investigate potential interactions between patients with implanted pacemakers and the S-ICD algorithm, and to check to what extent the manual screening tool—which was designed for detection of T-wave oversensing (TWOS)—can predict a safe combination of the devices. Of particular interest was the occurrence of oversensing; double, triple, and quadruple counting; and morphology changes induced with intermittently paced and non-paced rhythms.

## 2. Methods

This prospective observational study utilized a cohort of 74 patients ≥ 18 years of age with previously implanted single- or dual-chamber pacemakers with an intrinsic rhythm of >40 bpm, presenting in the Department of Cardiology at the University Hospital Giessen from March 2016 to October 2017. Patients with ongoing arrhythmia, such as atrial fibrillation or repetitive (supra-)ventricular complexes, were included in the analyses. All included patients had pacemakers implanted with the option of unipolar as well as bipolar pacing modes. All patients were enrolled during hospitalization. The right ventricular (RV) pacing lead was positioned either at the RV apex or the RV septum. Informed consent was obtained from all patients. The study was approved by the local research ethics committee (approval number: 83/15) and conformed to the ethical guidelines of the 1975 Declaration of Helsinki and its later amendments.

In the first step, surface rhythm strips (1-lead ECG) were recorded by the ZOOM^®^ LATITUDE^TM^ programming system, model 3120 (Boston Scientific, Marlborough, MA, USA), along all imaginary sensing vectors of the S-ICD system in the following pacemaker stimulation modes (if applicable due to basic rhythm), to screen for T-wave oversensing: intrinsic rhythm, VVI 3.0 V/0.5 ms; VVI 7.5 V/0.5 ms; DDD 3.0 V/0.5 ms; DDD 7.5 V/0.5 ms; AAI 3.0 V/0.5 ms; and AAI 7.5 V/0.5 ms (unipolar and bipolar).

**Figure 1:**
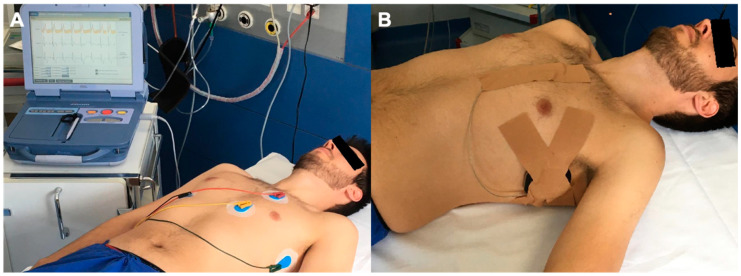
(**A**) Screening ECG with manufacturer’s recommended electrode placement. (**B**) Epicutaneous S-ICD dummy placement.

Corresponding to the regular location of an imaginary implanted S-ICD, surface skin electrodes were placed above the S-ICD can position (midaxillary line; 5/6th intercostal space), the proximal S-ICD sensing electrode (1 cm left lateral to the xiphoid process), and the distal S-ICD sensing electrode (14 cm cranial to the lower electrode). A ground electrode was placed on the right-lower abdomen (Figure 1A), as described previously [15]. Screening was performed in the supine position. The use of the manual screening tool (model 4744, Boston-Scientific) has previously been reported [16]. To ensure interobserver reliability for data analysis with the manual screening tool, all measurements were made by two assistant physicians (S.K. and T.G.), who analyzed the data independently. In cases where results were divergent, an expert electrophysiologist determined the eligibility. A patient was considered eligible if ≥1 vector was found without presumed TWOS.

The second step of the protocol was to fit an S-ICD to the patient epicutaneously, independent of the result of the manual screening tool (Figure 1B). The S-ICD and its lead were fixed by adhesive plaster, and electrical conductivity was enhanced by ultrasound gel on the S-ICD lead and device itself, to reduce electrical skin resistance.

To mimic the programming settings used in the UNTOUCHED trial for primary prevention [17], a virtual conditional shock zone was adopted to 200 bpm as well as a shock zone to 250 bpm (but not programmed), in order to prevent shock delivery. After acquisition of intrinsic rhythm, the same standardized protocol as that used for the screening tool was applied (pacing VVI, DDD, and AAI with uni- and bipolar stimulation (3.0 V/0.5 ms and 7.5 V/0.5 ms), 15 bpm faster than the underlying rhythm, but at least 90 bpm). In the DDD mode, the AV delay was set minus 20 ms of intrinsic conduction to ensure ventricular pacing. Every stimulation mode was recorded via S-ECG in every vector. The eligibility of the sensing vector of the previously implanted S-ICD system was confirmed when all consecutive QRS complexes were gapless—annotated as “S” (sense)—and if double counting was absent.

Statistical analyses were performed with GraphPad Prism 8.4.0 for macOS (GraphPad Software, LCC, San Diego, CA, USA). If a normal distribution of continuous variables was confirmed, data were given in mean values ± standard error of the mean (SEM). Comparisons between two groups were performed using the Student’s *t*-test. In the case of non-normal distributions, data were given as median values with 25th/75th interquartile ranges, and statistical comparisons were made using the Mann–Whitney rank sum test.

## 3. Results

A total of 74 patients were included consecutively in the study, of whom 31 (42.9%) were female (Table 1). The median age was 79 years, thereby presenting a typical pacemaker patient cohort with a normal left ventricular ejection fraction (LVEF). The main indications for pacemaker implantation were sick-sinus syndrome in 34 patients (45.9%), intermittent second-degree AV block in 9 patients (12.2%), and intermittent third-degree AV block in 16 patients (21.6%). Of the remaining patients, nine had bradycardic atrial fibrillation (12.1%), one had hypersensitive carotid sinus syndrome (1.4%), three had trifascicular bundle branch block with syncope, and two patients received a pacemaker due to cardioinhibitory neurocardiogenic syncope (2.7%). The median duration from implantation of the pacemaker to the investigation date was 360 days. The site of implantation of the pacemaker was on the right pectoral infraclavicular side in 29 patients (39.2%) and on the left pectoral infraclavicular side in 44 patients (59.5%). One pacemaker was located intraabdominally with an epicardial lead. The RV lead position was apical in 21 patients (28.4%), mid-septal in 18 patients (24.3%), and at the high septal region/RVOT in 31 patients (41.9%). Seventeen patients (23%) suffered from chronic obstructive pulmonary disease (COPD), partly with concomitant lung emphysema. Fourteen patients (18.9%) had undergone major cardiothoracic surgery with median sternotomy. Further baseline characteristics are provided in Table 1.

**Figure 2:**
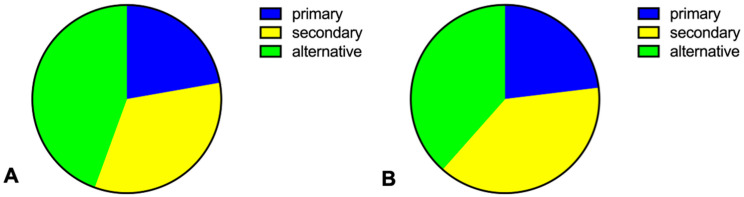
**Distribution of vectors without evidence of TWOS on ECG using manual screening tool.** (**A**) Analysis of constant possible vectors considering all pacing modes (uni- and bipolar) using the manual screening tool. (**B**) Analysis of constant possible vectors using the manual screening tool considering only bipolar pacing.

All patients were investigated in the VVI pacing mode, with 64.9% in the DDD mode and 48.6% in the AAI mode. A total of 2118 vectors were obtained across 74 patients, including the intrinsic pacing mode and all available pacing modes in uni- and bipolar pacing with different amplitudes (3.0 V/0.5 ms and 7.5 V/0.5 ms). Using the manual screening tool, 94.6% of our patient cohort showed eligibility in the intrinsic rhythm. Atrial pacing showed much higher eligibility (85.7–91.7%) than the VVI or DDD pacing modes (43.8–60.4%) (Table 2). However, when ≥1 constant vectors were required in intrinsic as well as in all pacing modes without under-, over-, or missensing, only 16 patients showed eligibility (21.6%) (Figure 2). If one takes the manufacturer’s recommendation that all pacemakers should be programmed in the bipolar stimulation mode into consideration, a total of 23 patients showed eligibility (31.1%). Only two patients were eligible in two vectors, and none were eligible in all three vectors. The alternative vector was estimated to be the most reliable in terms of preventing TWOS according to the manual screening tool (alternative vector 44.4% vs. secondary vector 33.3% vs. primary vector 22.2%; Figure 2A). When the results of unipolar pacing were excluded, the alternative and secondary vector showed equal results in bipolar stimulation only (alternative vector 38.5% vs. secondary vector 38.5% vs. primary vector 23%; Figure 2B).

**Figure 3:**
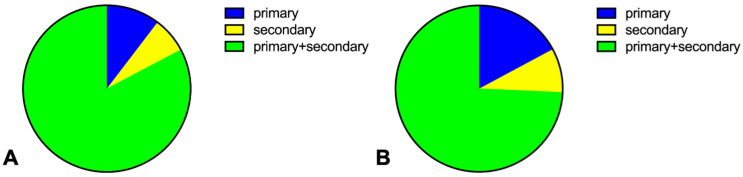
**Distribution of vectors without evidence of oversensing or noise on S-ECG using an epicutaneously placed S-ICD.** (**A**) Analysis of constant possible vectors considering all pacing modes (uni- and bipolar) using an epicutaneous S-ICD. (**B**) Analysis of constant possible vectors using an epicutaneous S-ICD considering only bipolar pacing.

Actual eligibility was reevaluated once with the epicutaneous S-ICD, irrespective of the previously obtained results from the manual screening tool. Patients were regarded as eligible if one vector was found without oversensing, noise (undersensing), or missensing. Though intrinsic rhythm showed similar values, with 94.6% eligibility compared with the manual screening tool, significant differences became obvious when the different pacing modes were considered (Table 3). Except for DDD unipolar pacing with low amplitude and AAI unipolar pacing with high amplitude, the manual screening tool underestimated eligibility by an absolute value of up to 40%, resulting in a low specificity for the screening tool in paced rhythms. Overall, eligibility was high (around 90%), especially in the bipolar pacing mode. The lowest eligibility was found in DDD unipolar stimulation and AAI unipolar pacing with high amplitude. A total of 60 patients (81.1%) were eligible in constant vectors in all pacing modes, including the intrinsic mode. Notably, the alternative vector was never an option due to noise or undersensing. Among the eligible patients, 58 patients (96.7%) were suitable in the bipolar pacing mode, 35 patients (58.3%) were suitable in uni- and bipolar pacing modes, and only two patients were only eligible in the unipolar pacing mode with a secondary vector. In most cases primary as well as secondary vectors were available. The minority of patients for whom only one vector was available showed no significant differences between the primary and secondary vector (Figure 3).

**Figure 4:**
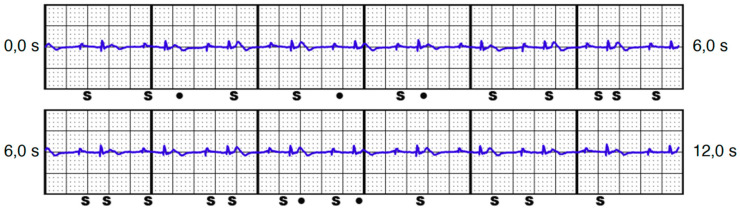
S-ECG with changing interpretation of stimulated atrial spike, ventricular spike, and QRS complex with T-wave, leading to oversensing (DDD unipolar pacing mode 3.0 V, secondary vector).

Oversensing could potentially occur due to stimulated spikes (atrial and/or ventricular) and the T-wave. Overall, including the QRS complex (which is meant to be sensed), this can potentially result in double, triple, or quadruple counting (e.g., see Figure 4). The intrinsic rhythm rarely showed oversensing (8.1%), whereas unipolar stimulation was more often associated with oversensing than bipolar stimulation (Table 3). DDD stimulation was the most common mode to trigger oversensing, followed by AAI stimulation. Oversensing was not necessarily associated with the amplitude under stimulation; on the contrary, in DDD and VVI stimulation, a lower amplitude led to more oversensing episodes (Table 4).

**Figure 5:**
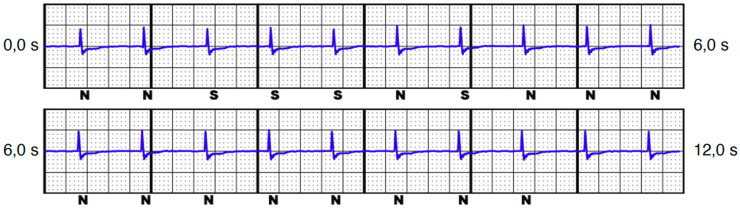
Example of a S-ECG with changing interpretation (noise or sense) of a stimulated spike in AAI pacing mode in lack of a visible QRS complex in the alternative vector.

Ultimately, a total of 16 patients (21.6%) with 31 vectors were identified who would have faced a triggered VT/VF episode in ≥1 vector (Table 5). While intrinsic rhythm was not associated with inadequate VT/VF detection, it proved to be a serious issue under pacemaker stimulation. Inadequate VT/VF detection was more likely to be triggered under the unipolar pacing mode, but it was also documented in three patients (4.1%) under the bipolar pacing mode, although this was in the VVI and DDD pacing modes only. All patients with VT/VF episodes under bipolar pacing also showed the same device–device interference in the unipolar pacing mode. While the alternative vector was never associated with VT/VF triggered episodes, the primary and secondary vectors were equally distributed (14 vs. 17 episodes). Most missensed VT/VF episodes occurred in the AAI pacing mode, followed by DDD and VVI modes. An example of such S-ECGs is illustrated in Figure 5.

**Figure 6:**
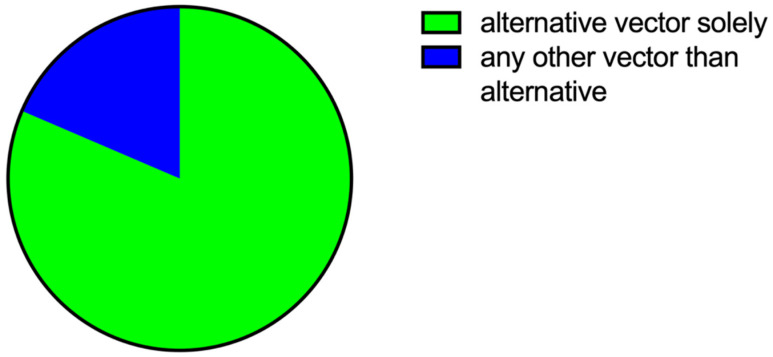
Noise detection or undersensing according to the vector configuration.

On the other hand, undersensing or the detection of a “noise” in ≥1 vector is a frequent phenomenon (Table 6). Interestingly, in most cases, detection of noise or undersensing occurred more often in the bipolar pacing mode. Moreover, detection of noise was not associated with the pulse amplitude, with the exception of the DDD unipolar pacing mode. Maximum episodes with noise or undersensing were detected in the intrinsic rhythm in ≥1 vector (90.5%). If one takes a closer look at the vectors detecting episodes with undersensing or noise, it can be seen to occur most frequently in the alternative vector (81.4%), making it a rare option for adequate sensing (Figure 6). In the alternative vector, the stimulation spike often showed a higher amplitude than the QRS complex (Figure 5).

In light of the above results, a total of 60 patients (81.1%) were considered eligible after investigation with the S-ICD dummy in at least one pacing mode and one vector, including the intrinsic rhythm with no signs of oversensing, missensing, or noise. Of this patient cohort, 58 patients (96.7%) were interference-free in the bipolar pacing mode and, interestingly, 2 patients (3.3%) were so in the unipolar pacing mode. Thirty-six patients (60%) were interference-free in unipolar and bipolar pacing modes. The alternative vector was never an option, mostly due to noise. The primary vector in 52 patients (86.7%) and the secondary vector in 50 patients (83.3%) showed smooth operation. In 42 patients (70%), the primary and secondary vector performed equally.

**Figure 7:**
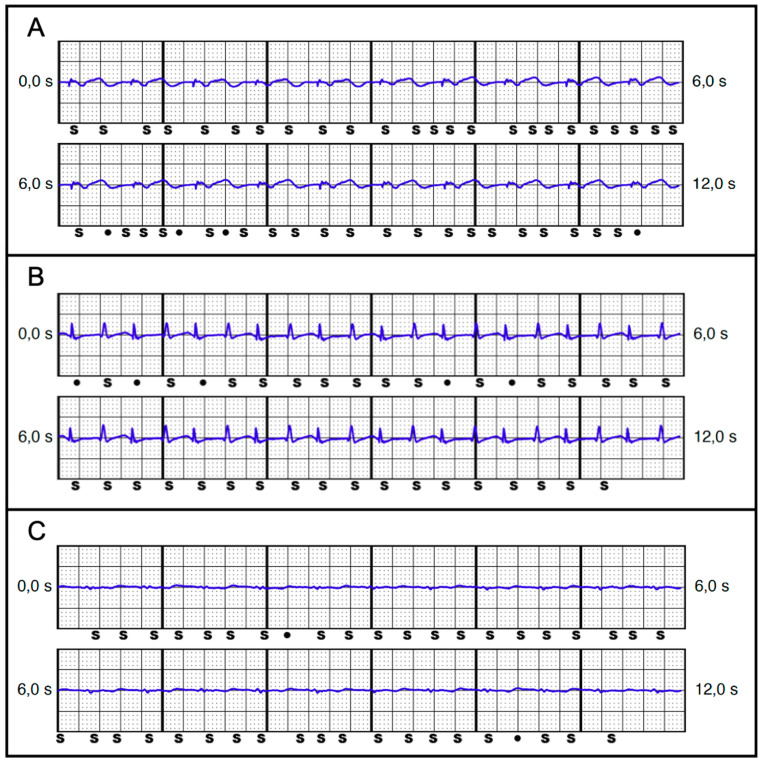
Examples of S-ECGs with missensing and thus potentially triggered inadequate shock delivery. (**A**) VVI unipolar pacing 3.0 V, primary vector. (**B**) AAI unipolar pacing 7.5 V, primary vector. (**C**) DDD bipolar pacing 3.0 V, secondary vector.

In comparing patients with missensing—and thus potentially triggered VT/VF episodes—(Figure 7) with the rest of the cohort (Table 7), we observed no significant differences in sex, (56.3% vs. 37.9% female; *p* = 0.25), age (76 vs. 79 years; *p* = 0.17), or BMI (25.2 vs. 27.1; *p* = 0.39). The fact that all patients with potential missensed episodes had dual-chamber pacemakers might be misleading, since VVI pacing was also responsible in some cases for missensing. All patients with VVI missensed episodes also showed missensing in DDD and/or AAI pacing modes. The difference in RV lead position was not relevant between the groups. The pacemaker implantation site was also not a significant factor. Considering comorbidities affecting the thoracic shape and integrity, we determined that COPD or lung emphysema were much more frequent in patients with missensed episodes than in the remainder (50% vs. 15.5%; *p* = 0.007). No differences were found in patients with prior cardiac surgery, including median sternotomy (12.5% vs. 20.7%; *p* = 0.51). A deeper analysis of the S-ECG revealed that patients with missensed episodes had a lower QRS amplitude in intrinsic rhythms (1.61 mV vs. 1.9 mV; *p* = 0.048) as well as under stimulation (1.03 mV vs. 1.3 mV; *p* < 0.0001). No significant differences were found between the amplitude of the stimulated QRS spikes (0.47 mV vs. 0.32 mV; *p* = 0.12), showing at most a tendency for patients with potentially triggered episodes to have a higher amplitude.

## 4. Discussion

In cases where two or more electrically active devices are used simultaneously in one patient—such as pacemakers, S-ICDs, cardiac contractility modulation, baroreceptor stimulator, WCDs, or other types of electrical devices—there is always a potential risk that the devices could interfere with each other, causing false detection with inadequate therapy delivery or even preventing appropriate therapy delivery. Although this principle is generally known to physicians, especially to those who focus on arrhythmias, data on device–device interactions are often scarce, especially when new devices are introduced to the market. Considering the prevalence of previously observed inadequate shock delivery by S-ICDs, which ranges from 4% [18] to 25% [19] due to various reasons, there is a clear need for the thorough investigation of possible over-, under- (noise), or missensing when interacting with a pacemaker.

In our study, we investigated a classic cohort of pacemaker recipients with typical baseline characteristics. We were able to study different pacemaker implantation sites, including the abdominal location, as well as different RV lead positions, including the epicardial position. Our results revealed that the manual screening tool does not sufficiently predict eligibility of the S-ICD combined with a pacemaker; thus, it lacks specificity. When compared with the epicutaneous screening test using our dummy, the actual eligibility rose from 21.6% to 81.1%, rendering the manual screening tool unnecessary in this context. Moreover, the manual screening tool is not designed for screening of undersensing or noise by the S-ICD. Notably, the alternative vector was never an option due to noise or undersensing.

Interestingly, two patients were only eligible in the unipolar pacing mode. It appears that in rare cases the paced spike helps the S-ICD to identify the QRS complex, although most of the time it is a risk for oversensing, which is especially documented in AAI and DDD pacing modes.

Inadequate VT/VF detection was more likely to be triggered with the unipolar pacing mode but was also documented with the bipolar pacing mode, although only in VVI and DDD pacing modes. This emphasizes the fact that the manufacturer’s recommendation for bipolar stimulation does not guarantee the total absence of over- and missensing, leading to inadequate shocks. Interestingly, most of the oversensing episodes were produced with the AAI pacing mode. Apparently, the INSIGHT algorithm is more successful in integrating the ventricular pacing spike into the QRS complex. Only a total of 78.4% of all patients were found to be compatible, with no evidence of electrical interference, which emphasizes the need for thorough testing.

Potential risk factors were identified that might increase the risk of missensing, including a dual-chamber pacemaker, COPD/lung emphysema, and a low intrinsic and stimulated QRS amplitude.

Obviously, a dual-chamber pacemaker is a risk factor for missensing compared to a single-chamber pacemaker because it tests up to two additional pacing modes (AAI + DDD), potentially tripling the risk of misinterpreted pacing spikes. Patients with cardiac resynchronisation therapy pacemakers were not included in our cohort but have the potential to be highly susceptible to misdetection by the S-ICD, particularly with significant time delays in biventricular pacing.

COPD and lung emphysema can change the configuration of the thorax as well as the electrical axis of the heart, which is of utmost importance when considering the implantation of an extrathoracic device such as an ICD. Patients with COPD and lung emphysema have also been linked with findings such as a lower QRS amplitude of the limb- and precordial leads [20].

The presence of a history of sternotomy with residual cerclage had no effect on potential triggering episodes, although slightly altered thoracic anatomy can be assumed, making the low QRS amplitude the biggest suspect in terms of risk factors. With these findings in mind, it is not surprising that we identified low QRS amplitude in the intrinsic rhythm—and even more so in the paced rhythm—as the most significant risk factors, as they make it more difficult for the INSIGHT algorithm to distinguish between the pacemaker spike and the true QRS. Remarkably, the sole amplitude of the stimulation spike was not significantly higher in the patient group with triggered episodes. In fact, specifically in the alternative vector, a high amplitude of the pacing spike, compared with a low amplitude of the QRS, leads to noise (see Figure 5).

Of course, many issues and doubts concerning interactions could be solved if the S-ICD were able to communicate with the pacemaker. Development of this technology is in progress, and we may have new options for our patients in the future, with a leadless pacemaker that is also able to deliver anti-tachycardia pacing therapy [21].

## 5. Conclusions

The combination of an S-ICD and a pacemaker may lead to inadequate shock delivery due to oversensing of pacing spikes, T-waves, and deformed QRS complexes, even if bipolar stimulation is programmed. Oversensing and pacemaker interactions cannot be adequately predicted by the manual screening tool when used in pacemaker patients. Thus, epicutaneous testing with a functional S-ICD dummy in all vectors and stimulation settings should be mandatory before considering implantation of an S-ICD in pacemaker patients.

## 6. Limitations

This is a prospective observational study. Although it was not possible to perform proper randomization, we at least had some control within the cohort itself by comparing intrinsic rhythms with paced rhythms.

Based on the baseline characteristics, this is a classic pacemaker cohort. Patients considered for S-ICD are typically much younger and may have different cardiac functions in terms of lower LVEF and an increased left ventricular end-diastolic volume, and possibly a higher BMI.

The applicability of our data from an epicutaneous S-ICD in our setup to an in-situ implanted S-ICD may be debatable, but to our knowledge this is the closest non-invasive screening approach available, especially in terms of ethical acceptability. This is why the S-ICD distributor, Boston-Scientific, routinely uses this method.

The antitachycardia therapy of the epicutaneously placed S-ICD was disabled to prevent inappropriate shocks to the patient, so we can only infer the risk of inappropriate therapy in real life from the level of oversensing observations.

## Figures and Tables

**Table 1:** Baseline characteristics.

Median Age (Years)	79 (74; 84)
Sex, *n* (%) Male Female	43 (58.1%) 31 (41.9%)
Mean body mass index (BMI) kg/m^2^	26.8 ± 0.6
Median LVEF (%)	60 (55; 65)
Indications for pacemaker, *n* (%) sick sinus/brady-tachy syndrome AV block II AV block III trifascicular block with syncope hypersensitive carotid sinus syndrome bradycardic atrial fibrillation cardioinhibitory syncope	34 (45.9%) 9 (12.2%) 16 (21.6%) 3 (4%) 1 (1.4%) 9 (12.1%) 2 (2.7%)
Median time since implantation of pacemaker (days)	360 (2; 1258)
Manufacturer, *n* (%) Abbott Biotronik Boston Scientific Medtronic	13 (17.6%) 55 (74.3%) 4 (5.4%) 2 (2.7%)
Type of pacemaker, *n* (%) single-chamber (VVI) dual-chamber (DDD)	15 (20.3%) 59 (79.7%)
Pacemaker implantation site, *n* (%) left infraclavicular right infraclavicular abdominal	44 (59.5) 29 (39.2%) 1 (1.4%)
RV lead position, *n* (%) RVOT/high septal mid-septal low septal apical epicardia	31 (41.9%) 18 (24.3%) 3 (4.1%) 21 (28.4%) 1 (1.4%)
Chronic obstructive lung disease/lung emphysema, *n* (%)	17 (23%)
Prior cardiac surgery, median sternotomy	14 (18.9%)

**Table 2:** Screening eligibility with the manual screening tool requiring ≥1 vectors. UP, unipolar; BP, bipolar.

Screening Elegibility with the Manual Screening Tool (1 out of 3 Vectors)													
Pacing Mode	Intrinsic	VVI (74/74)				DDD (48/74)				AAI (36/74)			
Amplitude (V)		3.0 UP	3.0 BP	7.5 UP	7.5 BP	3.0 UP	3.0 BP	7.5 UP	7.5 BP	3.0 UP	3.0 BP	7.5 UP	7.5 BP
Eligibility (%)	94.6	44.6	51.4	54.1	50.0	60.4	56.3	43.8	56.3	88.9	85.7	86.1	91.7

**Table 3:** Eligibility under S-ICD testing requiring ≥1 vectors. UP, unipolar; BP, bipolar; exclusion of oversensing, noise (undersensing), and missensing.

Screening Elegibility with the S-ICD (1 out of 3 Vectors)													
Pacing Mode	Intrinsic	VVI (74/74)				DDD (48/74)				AAI (36/74)			
Amplitude (V)		3.0 UP	3.0 BP	7.5 UP	7.5 BP	3.0 UP	3.0 BP	7.5 UP	7.5 BP	3.0 UP	3.0 BP	7.5 UP	7.5 BP
Eligibility (%)	94.6	91.9	87.8	79.7	90.4	31.3	93.8	45.8	93.8	88.9	97.2	58.3	94.4

**Table 4:** Oversensing (double, triple, or quadruple sensing without VT/VF triggering) in ≥1 vector.

Oversensing (Double, Triple, or Quadruple Sensing without VT/VF-Triggering) in ≥1 Vector													
Pacing Mode	Intrinsic	VVI (74/74)				DDD (48/74)				AAI (36/74)			
Amplitude (V)		3.0 UP	3.0 BP	7.5 UP	7.5 BP	3.0 UP	3.0 BP	7.5 UP	7.5 BP	3.0 UP	3.0 BP	7.5 UP	7.5 BP
Oversensing (%)	8.1%	27.0	16.2	8.1	12.2	50.0	10.4	33.3	14.6	33.3	8.3	50.0	8.3

**Table 5:** Missensed VT/VF detection in ≥1 vector.

Missensed VT/VF or Detection in ≥1 Vector 68/74													
Pacing Mode	Intrinsic	VVI (74/74)				DDD (48/74)				AAI (36/74)			
Amplitude (V)		3.0 UP	3.0 BP	7.5 UP	7.5 BP	3.0 UP	3.0 BP	7.5 UP	7.5 BP	3.0 UP	3.0 BP	7.5 UP	7.5 BP
Triggered VT/VF (%)	0%	4.1	4.1	1.4	1.4	10.4	4.2	8.3	6.3	11.1	0	25.0	0

**Table 6:** Undersensing or detection of “noise” in ≥1 vectors.

Undersensing or Detection of “Noise” in ≥1 Vector 68/74													
Pacing Mode	Intrinsic	VVI (74/74)				DDD (48/74)				AAI (36/74)			
Amplitude (V)		3.0 UP	3.0 BP	7.5 UP	7.5 BP	3.0 UP	3.0 BP	7.5 UP	7.5 BP	3.0 UP	3.0 BP	7.5 UP	7.5 BP
Undersensing/Noise (%)	90.5%	44.6	87.8	40.5	82.4	35.1	59.5	48.7	58.1	35.1	44.6	17.6	43.2

**Table 7:** Characteristics of patients and pacemaker systems with triggered VT/VF episodes compared with total patient cohort.

Parameter	Triggered (16 Patients)	Not Triggered (58 Patients)	*p*-Value
Sex, *n* (%) Male Female	7 (43.7%) 9 (56.3%)	36 (62.1%) 22 (37.9%)	0.25
Median age (years)	76 (81; 90)	79 (74; 84)	0.17
Mean body mass index (BMI) kg/m^2^	25.2 ± 1.6	27.1 ± 0.6	0.39
Type of pacemaker, *n* (%) single-chamber dual-chamber	0 16 (100%)	15 (25.9%) 43 (74.1%)	0.03
Pacemaker implantation site, *n* (%) left infraclavicular right infraclavicular abdominal	12 (75%) 4 (25%) 0	32 (55.2) 25 (43.1%) 1 (1.7%)	0.25
RV lead position, *n* (%) RVOT/high-septal mid-septal low septal apical epicardial	9 (56.3%) 3 (18.7%) 0 4 (25%) 0	22 (37.9%) 15 (25.9%) 3 (5.2%) 17 (29.3%) 1 (1.7%)	0.25 0.70 0.77
Chronic obstructive lung disease/lung emphysema, *n* (%)	8 (50%)	9 (15.5%)	0.007
Prior cardiac surgery, median sternotomy	2 (12.5%)	12 (20.7%)	0.51
Intrinsic QRS amplitude	1.61 ± 0.1	1.9 ± 0.1	0.048
Stimulated QRS amplitude (S-ECG), mV	1.03 ± 0.04	1.30 ± 0.03	<0.0001
Stimulated spike amplitude (S-ECG), mV	0.47 ± 0.03	0.32 ± 0.01	0.12

## Data Availability

Data available on request due to privacy restrictions. The data presented in this study are available on request from the corresponding author.
